# Antibiotic resistance and draft genome profiles of 10 *Streptococcus pneumoniae* and 3 *Streptococcus pseudopneumoniae* strains isolated from the nasopharynx of people living with human immunodeficiency virus in Ghana

**DOI:** 10.1128/mra.00505-24

**Published:** 2024-10-04

**Authors:** Lawrencia Ami Emefa Ativi, Mildred Adusei-Poku, William Boateng, Christian Owusu-Nyantantakyi, Justice Kwesi Danso, Agnes Oclu, Alfred Bortey, Grebstad Rabbi Amuasi, Blessing Kofi Adu Tabi, Elijah Paintsil, Kwasi Torpey, Nicholas Dzifa Dayie, Beverly Egyir

**Affiliations:** 1 Department of Medical Microbiology, University of Ghana Medical School, Accra, Ghana; 2 Department of Bacteriology, Noguchi Memorial Institute for Medical Research, Accra, Ghana; 3 Department of Pediatrics, Yale School of Medicine, Yale University, New Haven, Connecticut, USA; 4 Department of Population, Family and Reproductive Health, School of Public Health, University of Ghana, Accra, Ghana; Loyola University Chicago, Chicago, Illinois, USA

**Keywords:** Africa, *Streptococcus pneumoniae*, *S. pseudopneumoniae*

## Abstract

Genomic data on clinically important bacteria species such as *Streptococcus pneumoniae* and *Streptococcus pseudopneumoniae* from low- and middle-income countries, including Ghana, are scarce. In this study, we provide data on antimicrobial resistance (AMR) and whole-genome profiles of a collection of streptococci species to support AMR surveillance efforts in the country.

## ANNOUNCEMENT

Streptococci are a major cause of a wide range of diseases including pneumoniae, bacteremia, and meningitis (1). The similarities between *Streptococcus pseudopneumoniae* and *Streptococcus pneumoniae* make distinguishing them using traditional typing methods challenging (2, 3). Information on the epidemiology and genomic characteristics of *S. pseudopneumoniae* is scarce in sub-Saharan countries, including Ghana. Like *S. pneumoniae*, *S. pseudopneumoniae* is frequently associated with high rates of antimicrobial resistance, particularly to penicillin, macrolides, co-trimoxazole, and tetracycline (3). This article details the antimicrobial susceptibility and genomic profiles of 10 *S. pneumoniae* and 3 *S*. *pseudopneumoniae* isolates recovered from nasopharyngeal swabs collected from HIV-positive individuals during a cross-sectional study conducted at three hospitals in the Greater Accra Region of Ghana: University of Ghana Hospital, LEKMA Hospital, and Korle Bu Teaching Hospital. The study received approval from the University of Ghana, College of Health Sciences Ethical and Protocol Review Committee (Protocol number: CHS-Et/M.7-P 4.3/2022–2023). The nasopharyngeal swab samples were plated on 5% sheep blood agar (Oxoid) and incubated anaerobically for 24 hours at 37°C. Bacteria identification was done using the matrix-assisted laser desorption/ionization-time-of-flight mass spectrometer. Antimicrobial susceptibility testing was performed on MH agar with 5% sheep blood using seven antibiotics (chloramphenicol, clindamycin, erythromycin, linezolid, tetracycline, trimethoprim-sulfamethoxazole, and vancomycin) in a disk diffusion assay and interpreted according to the Clinical Laboratory Standards Institute guidelines (CLSI, 2023). *S. pneumoniae* ATCC 49619 strain was used as control.

Following the manufacturer’s instructions, DNA was extracted from pure overnight cultures using the QIAamp DNA Mini Kit (Qiagen, Germany) after suspending the colonies in lysozyme. The extracted DNA was then quantified using the Qubit 4.0 fluorometer and the High Sensitivity dsDNA Assay Kit. Libraries were generated using the Illumina DNA library preparation kit (Illumina Inc., San Diego, CA, USA). The quality and concentration of the libraries were subsequently assessed using the 2100 bioanalyzer system (Agilent) and Qubit 4.0 fluorometer, respectively. The libraries underwent dilution to reach a concentration of 2 nM, subsequently pooled, and subjected to sequencing on the Illumina Miseq platform (Illumina Inc., San Diego, CA) using a 2 × 300 bp chemistry. After sequencing, Trimmomatic v.0.39 (4) was employed in the trimming of adaptors and reads with a quality score below 20. FastQC v.1.0 (https://www.bioinformatics.babraham.ac.uk) was used in the quality control checks of the reads. Unicycler v.0.5.0 (5) was used in the assembly of the trimmed reads and subsequently assessed using Quast v.5.2.0 (6). All genomes had a Q-score greater than 30, a minimum coverage of 40×, less than 300 contigs, and a minimum contig size exceeding 200 bp. Post assembly, KmerFinder v.4.1 (https://cge.food.dtu.dk/services/KmerFinder/) (7) was employed in bacterial species identification. ResFinder v.4.1 (https://cge.food.dtu.dk/services/ResFinder/) (8), CARD v.3.2.9 (https://card.mcmaster.ca/) (9), VirulenceFinder v.2.0 (https://cge.food.dtu.dk/services/VirulenceFinder/) (10), and MLST v.2.0 (https://cge.food.dtu.dk/services/MLST/) (11) were used in the determination of sequence types, resistance, and virulence genes using default settings. The assembled genomes were annotated using Prokka v.1.14.6 (12).

**TABLE 1:** Antimicrobial resistance profiles and genomic characteristics of isolates^*a*^

Isolate ID	Strain	Hospital	Genome accession no.	SRA accession no.	Biosample accession no.	Antibiotic resistance profile	Resistance genes	Virulence genes	Sequence type	Coverage (×)	Genome size (bp)	No. of contigs	N50 (bp)	GC content (%)	No. of reads	No. of CDS	No. of genes	No. of rRNA	No. of tRNA	No. of tmRNA
*S. pneumoniae*																				
HRV-23–022	SA1	LEKMA	JARUNO000000000	SRR24071752	SAMN34074645	ERY, CLI, TE, and SXT	*erm(B*), *mef(A*), *msr(D*), *tet(M*), *patA*, *patB*, *pmrA*, and *VanY*	*cbpD*, *cbpG*, *lytA*, *lytB*, *lytC*, *pce/cbpE*, *pspA*, *pspC/cbpA*, *pavA*, *lmb*, *srtA*, *rrgA*, *plr/gapA*, *rrgB*, *rrgC*, *srtB*, *srtC*, *srtD*, *hysA*, *nanA*, *eno*, *piaA*, *piuA*, *psaA*, *cppA*, *iga*, *htrA/degP*, *tig/ropA*, *zmpB*, and *ply*	ST320	84	2,048,242	77	71,641	39.7	1,102,816	1,994	2,033	3	35	1
HRV-23-D44	SA3	LEKMA	JARUNM000000000	SRR24071750	SAMN34074647	SXT	*patA*, *patB*, *pmrA*, and *VanY*	*cbpD*, *cbpG*, *lytB*, *lytC*, *pce/cbpE*, *pspA*, *pspC/cbpA*, *pavA*, *lmb*, *srtA*, *slrA*, *plr/gapA*, *cla*, *nanA*, *eno*, *piaA*, *piuA*, *psaA*, *cppA*, *iga*, *htrA/degP*, *tig/ropA*, *zmpB*, and *ply*	ST17228	104	2,149,241	75	89,076	39.4	1,448,104	2,127	2,167	3	36	1
HRV-23–111	SA4	KBTH	JBBKAG000000000	SRR28419022	SAMN40566640	TE and SXT	*patA*, *patB*, *pmrA*, and *tet(M*)	*cbpD*, *cps4A*, *cps4B*, *hysA*, *lytA*, *lytC*, *nanB*, *pavA*, *pce*, *pfbA*, *ply*, and *psaA*	ST172	47	2,135,800	82	52,858	39.5	498,944	2,110	2,149	3	35	1
HRV-23–147	SA5	LEKMA	JBBKAF000000000	SRR28419021	SAMN40566641	TE	*patA*, *patB*, *pmrA*, and *tet(M*)	*cbpD*, *cbpG*, *cps4A*, *cps4B*, *cps4C*, *cps4D*, *hysA*, *lytA*, *lytC*, *nanB*, *pavA*, *pce*, *pfbA*, *ply*, and *psaA*	ST18370	67	2,025,352	137	44,799	39.7	616,808	1,955	1,989	3	30	1
HRV-23–148	SA6	LEKMA	JBBKAE000000000	SRR28419019	SAMN40566642	C, TE, and SXT	*patA*, *patB*, *pmrA*, *cat(pC194*), and *tet(M*)	*cbpD*, *cps4A*, *cps4B*, *cps4C*, *cps4D*, *hysA*, *lytA*, *lytC*, *nanB*, *pavA*, *pce*, *ply*, *psaA*, *rrgA*, *rrgC*, *srtC-1/srtB*, *srtC-2/srtC*, and *srtC-3/srtD*	ST802	94	2,160,343	68	77,187	39.4	971,194	2,150	2,191	3	37	1
HRV-23–167	SA7	KBTH	JBBKAD000000000	SRR28419018	SAMN40566643	TE and SXT	*patA*, *patB*, *pmrA*, and *tet(M*)	*cbpD*, *cbpG*, *cps4A*, *cps4B*, *cps4C*, *cps4D*, *hysA*, *lytA*, *lytC*, *nanB*, *pavA*, *pce*, *pfbA*, *ply*, and *psaA*	ST18372	40	2,050,151	104	42,645	39.6	435,710	2,024	2,060	3	32	1
HRV-23–33	SA8	LEKMA	JBBKAC000000000	SRR28419017	SAMN40566644	C, TE, and SXT	*patA*, *patB*, *pmrA*, and *tet(M*)	*cbpD*, *cbpG*, *cps4A*, *cps4B*, *cps4C*, *cps4D*, *hysA*, *lytA*, *lytC*, *nanB*, *pavA*, *pce*, *pfbA*, *ply*, and *psaA*	ST18370	98	2,052,594	79	90,420	39.7	881,934	2,003	2,055	3	37	1
HRV-23–443	SA9	LEKMA	JBBKAB000000000	SRR28419016	SAMN40566645	TE and SXT	*patA*, *patB*, *pmrA*, and *tet(M*)	*cbpD*, *cbpG*, *hysA*, *lytA*, *lytB*, *lytC*, *nanB*, *pavA*, *pce*, *pfbA*, *ply*, and *psaA*	ST700	101	2,057,773	69	82,415	39.5	970,696	2,016	2,055	3	35	1
HRV-23–66	SA10	LEKMA	JBBKAA000000000	SRR28419015	SAMN40566646	C, TE, and SXT	*patA*, *patB*, *pmrA*, *cat(pC194*), and *tet(M*)	*cbpD*, *cbpG*, *hysA*, *lytA*, *lytC*, *nanB*, *pce*, *pfbA*, *ply*, *psaA*, and *pspC/cbpA*	ST13456	47	2,052,751	84	39,511	39.6	464,844	2,001	2,035	3	30	1
HRV-23–71	SA11	LEKMA	JBBJZZ000000000	SRR28419014	SAMN40566647	TE and SXT	*patA*, *patB*, *pmrA*, and *tet(M*)	*cbpD*, *cbpG*, *cps4A*, *cps4B*, *cps4C*, *cps4D*, *hysA*, *lytA*, *lytB*, *lytC*, *nanB*, *pavA*, *pce*, *pfbA*, *ply*, and *psaA*	ST18376	84	2,115,087	90	64,833	39.5	847,416	2,083	2,120	3	33	1
*S. pseudopneumoniae*																				
HRV-23–029	SA12	LEKMA	JBBJZY000000000	SRR28419013	SAMN40566648	ERY, CLI, and SXT	*patB*	*cbpD*, *lytA*, *lytC*, *pavA*, *pce*, *ply*, and *psaA*	UNK	46	1,941,672	47	75,591	40.3	387,928	1,769	1,795	2	25	1
HRV-23–34	SA13	LEKMA	JBBJZX000000000	SRR28419012	SAMN40566649	TE and SXT	*patA*, *patB*, *pmrA*, and *tet(M*)	*cbpD*, *hysA*, *lytC*, *pavA*, *pce*, and *psaA*	UNK	91	2,155,673	292	20,469	39.9	918,962	1,983	1,999	2	13	1
HRV-23–36	SA14	LEKMA	JBBJZW000000000	SRR28419020	SAMN40566650	TE and SXT	*hysA*, *lytA*, *lytC*, *pavA*, *pce*, *ply*, and *psaA*	*patA*, *patB*, *pmrA*, and *tet(M*)	UNK	135	2,057,522	45	131,504	40.0	1,208,424	1,876	1,917	2	38	1

^*a*^ ERY, erythromycin; CLI, clindamycin; TE, tetracycline; SXT, trimethoprim-sulfamethoxazole; C, chloramphenicol; UKN, unknown; LEKMA, LEKMA Hospital; KBTH, Korle Bu Teaching Hospital.

## Data Availability

The genomes were deposited in the National Center for Biotechnology Information database with BioProject number PRJNA952500. Table 1 summarizes the antibiotic profile and genomic characteristics of the isolates.
